# Current Approach to Complications and Difficulties during Transrectal Ultrasound-Guided Prostate Biopsies

**DOI:** 10.3390/jcm13020487

**Published:** 2024-01-16

**Authors:** Salloum Osama, Crenguta Serboiu, Iulian-Alexandru Taciuc, Emil Angelescu, Costin Petcu, Tiberiu Alexandru Priporeanu, Andreea Marinescu, Adrian Costache

**Affiliations:** 1 Pathology Department, Carol Davila University of Medicine and Pharmacy, 050096 Bucharest, Romania; os.salloum@gmail.com (S.O.); alexandertaciuc@gmail.com (I.-A.T.); adriancostacheeco@yahoo.com (A.C.); 2 Cellular Biology and Histology Department, Carol Davila University of Medicine and Pharmacy, 020021 Bucharest, Romania; 3 Urology Department, Carol Davila University of Medicine and Pharmacy, 022328 Bucharest, Romania; eangelescu1964@gmail.com (E.A.); tpriporeanu@gmail.com (T.A.P.); 4 Radiology and Imaging Department, Carol Davila University of Medicine and Pharmacy, 050095 Bucharest, Romania

**Keywords:** TRUS, prostate biopsy, urology, prostate cancer detection

## Abstract

Prostate cancer is one of the most common male malignancies worldwide. It affects middle-aged men (45–60 years) and is the leading cause of cancer-related mortality in Western countries. The TRUS (trans rectal ultrasound)-guided prostate biopsy has been a standard procedure in prostate cancer detection for more than thirty years, and it is recommended in male patients with an abnormal PSA (prostate-specific antigens) or abnormalities found during digital rectal examinations. During this procedure, urologists might encounter difficulties which may cause subsequent complications. This manuscript aims to present both the complications and the technical difficulties that may occur during TRUS-guided prostate biopsy, along with resolutions and solutions found in the specialized literature. The conclusions of this manuscript will note that the TRUS-guided prostate biopsy remains a solid, cost-efficient, and safe procedure with which to diagnose prostate cancer. The complications are usually self-limiting and do not require additional medical assistance. The difficulties posed by the procedure can be safely overcome if there are no other available alternatives. Open communication with the patients improves both pre- and post-procedure compliance.

## 1. Introduction

Prostate cancer is one of the most common male malignancies worldwide. It affects middle-aged men (45–60 years), and is the leading cause of cancer-related mortality in Western countries [1]. Overall, the incidence between 2014 and 2018 remained stable, with a 4–6% increase in advanced disease and an increase from 3.9% to 8.2% in advanced stages [2]. On average, there are 190,000 new cases of prostate cancer, and around 80,000 deaths each year, although the incidence differs between ethnic groups and geographical regions. The screening of prostate-specific antigens (PSA) plays a significant role in detecting cancer in its early stages, and because of this, the highest incidence rates are seen in developed countries [3]. As with any cancer, the success rate and the recurrence-free survival are strongly related to the stage of the disease, so it should be diagnosed as early as possible. For the early detection of low-volume prostate cancer, most countries use a combination of serum PSA level screening, along with a digital rectal examination and a systematic transrectal ultrasound-guided prostate biopsy [4]. Long-term studies have shown that PSA-based screening reduces the risk of mortality caused by prostate cancer, but there are still discussions on this topic because of the considerable number of overdiagnoses that can occur because of it [5,6].

A prostate biopsy is the basic investigation tool with which a certain diagnosis is made, and with the recent diagnostic revolution and advancing technology, the accuracy of this biopsy has increased even further. In recent years, the transrectal ultrasound (TRUS)-guided prostate biopsy with 12 cores has been considered the gold-standard procedure in prostate cancer detection [7]. The actual ability of the transrectal ultrasound to detect prostate cancer itself is limited, with a sensitivity of about 73.6% and a specificity of 61.3%. Thus, the diagnostic accuracy of prostate biopsies varies with the imaging techniques used [8]. The TRUS prostate biopsy, however, has been demonstrated to have a high specificity of 96% but a sensitivity of only 48% [9].

This study aims to centralize data regarding both the complications of TRUS-guided prostate biopsies and the procedural difficulties that may occur during this procedure.

## 2. Methods

**Figure 1:**
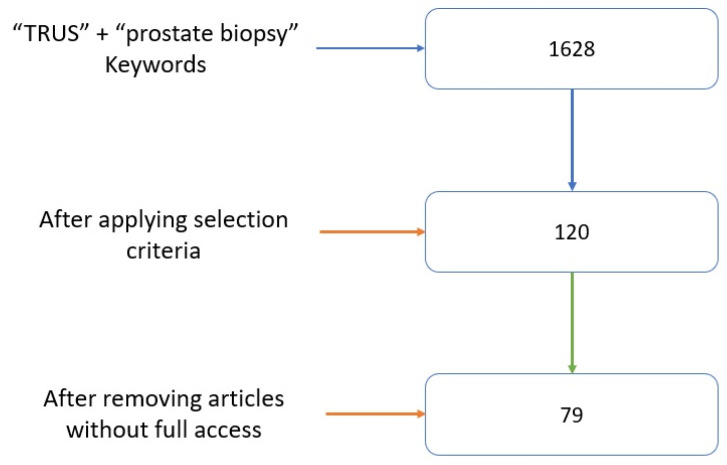
Article selection.

This manuscript is a narrative review of the literature, and the study was conducted in 2023, throughout the year. This manuscript was created using PubMed articles, with “TRUS” and “prostate biopsy” as the main search keywords. Some references were obtained using words from TRUS-guided prostate biopsy subfields, and comparisons with other biopsy techniques were made. Other references were obtained by cross-referencing key articles. Of the 1628 results returned by the PubMed platform, 79 articles were selected, and they adhered to the following criteria: must contain faithful information (complete articles, with realistic information and achievable results); must have access to the article; and must include information linked to TRUS-guided prostate biopsies. Articles with misleading titles and keywords were removed. With a few exceptions, the proposed references used in this article were published after 2013 (Figure 1).

## 3. Gleason Score

The Gleason scoring system was used as the prostate cancer aggressivity grading system. It was first developed by Donald Gleason in the 1960s, and it has been revised since then. The revised Gleason score aimed to improve the outcomes in all groups at risk of prostate cancer, and to reduce the overtreatment of indolent cancers. The Gleason score ranges between 1 and 5; these numbers describe whether the cell in the sample looks more like a well-differentiated or anaplastic cell. The two dominant grades of malignant cell are each given a score, and the sum of these scores describes the grade [10,11]. The ISUP score is a relatively new system that grades prostate cancer based on the Gleason score and cancer aggressiveness. The higher the grade, the more likely the cancer will spread [12]. To make a diagnosis, it is very important to have suspicions. In the specialized literature, several correlations have been made between the diagnostic accuracy and the ISUP score. Using a multiparametric MRI, some prostate cancers can be detected at ISUP grade of 2, where certain lesions can be missed or overlooked during TRUS-guided prostate biopsy [13].

## 4. Transrectal Ultrasound (TRUS)-Guided Prostate Biopsy

**Figure 2:**
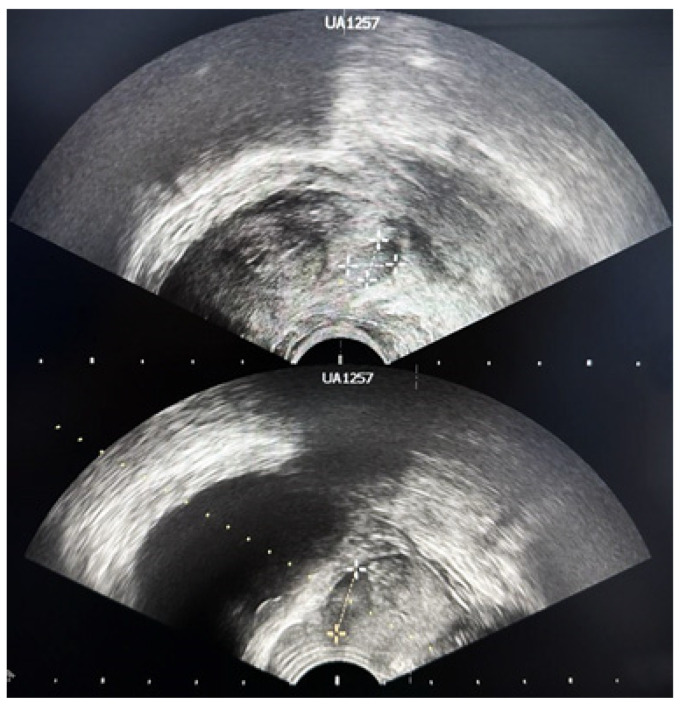
Prostatic nodule (6 mm) located at the base of the prostate in a patient with a PSA value of 12.3 ng/mL.

TRUS is a fast procedure that uses the ultrasound waves to visually analyze the prostate gland. Before investigation, the patient may have an enema (to clear out any feces or gas which could interfere). The patient is placed on his left side with his knees pulled up towards his chest and a small probe is placed into the patient’s rectum. The imaging instigation usually starts at the base of the bladder and then, by rotating the probe a full picture of the prostate can be acquired (Figure 2) [12,14]. Sometimes the prostate tumors create specific echoes that are different from normal prostate tissue. TRUS alone cannot detect all prostate tumors, but it can detect some tumors that are not felt by the physician during the digital rectum examination. The isoechoic areas represent the normal tissue. The hyperechoic areas often indicate the presence of calcifications, while the hypoechoic areas are usually associated with cancer [15]. The new Prostate Imaging Reporting and Data System version (PI-RADSv2) [16] has improved the PI-RADS 4 and 5 lesion detection during TRUS [17,18].

The TRUS-guided prostate biopsy has been a standard procedure for prostate cancer detection for more than thirty years and it is recommended in male patients with abnormal PSA, or an abnormality felt on a digital rectal examination [19]. In the systematic prostate biopsy, the patient is prepared as for TRUS, but the doctor will usually inject a local anesthetic into the rectum. Other types of anesthesia may be used (they will be discussed in the Section 6.6 “Pain”). A core-biopsy needle is then pushed along the ultrasound probe into the prostate gland. The recommended number of cores sampled is around 10–12 per patient [20,21]. A study published in 2015 compared three groups of patients who had 6, 12, and 18 core biopsies and the results showed a significant difference between the 6 and 12 core biopsies groups, as there were more cases of prostate cancer detected in the last one; and no significant difference between the 12 and 18 core biopsies groups. As a result, 12 core biopsies proved to be the optimal number for sampling [22].

## 5. Post-Procedural Complications

**Figure 3:**
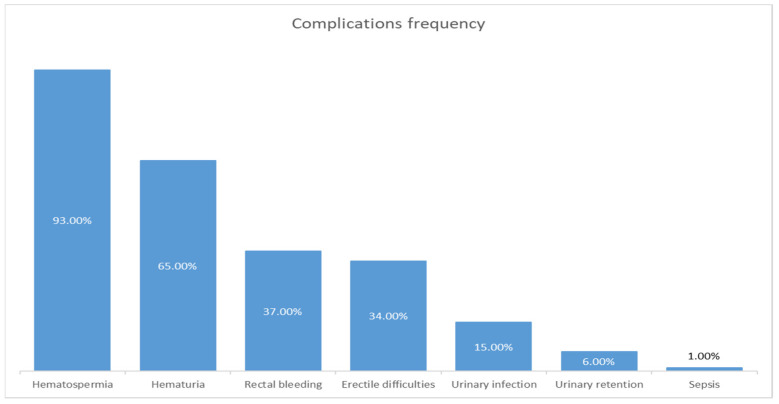
Bar chart depicting the main complications based on their frequency (data extracted from articles used in this study).

The TRUS biopsy has low rates of severe complications, although there remains room for improvement in current practice to improve the tolerability and reduce the incidence of post-biopsy infection [19]. The complications may include urethral bleeding, hematospermia, rectal bleeding, urinary retention, urinary tract infection, sepsis, erectile difficulties, and pain. Most of the complications post-biopsy are often self-limiting, even though they are frequent [23,24]. The frequency of the complications can vary from study to study, although the trend remains the same. In Figure 3, the chart presents the complications’ frequency ordered from highest to lowest. The percentages were taken from various studies that will be mentioned in the next sub-chapters.

### 5.1. Hematospermia

The presence of blood in the ejaculation liquid is the most frequent reported complication after prostate biopsy, in some studies reaching an incidence of up to 93% of patients. In the UK, there was a large prospective study which reported hematospermia in 92.6% of patients. The symptoms have been reported within 35 days after the biopsy. A percent of 26.6% of patients have perceived this as a moderate/serious problem. This high percentage might also be influenced because of the triggered anxiety [25,26]. Another retrospective study has also revealed that hematospermia was the most reported complication after TRUS-guided prostate biopsy, with 36.3% of patients affected by it, but more than this, the study revealed a strong correlation between hematospermia and the number of cores taken (the frequency increased with more cores taken) [27].

### 5.2. Hematuria

Hematuria is the second most frequent reported complication of prostate biopsy according to some studies. A large prospective study (including 1147 male patients) has showed that after TRUS-guided biopsy, 65.8% of patients reported hematuria within 35 days from procedure. In comparison with hematospermia, only 6.2% of them considered the symptoms moderate/severe [25]. As with any other bleeding complication, the frequency will greatly vary with patient-related factors: prostate volume, medical comorbidities, the use of anticoagulant medications, and presence of coagulopathies [24]. A study on bleeding complications comparing six, eight, and twelve core samplings did not find any significant differences in the prevalence of hematuria by the TRUS-guided prostate biopsy number of cores [28].

### 5.3. Rectal Bleeding

Rectal bleeding is also a common bleeding complication in TRUS-guided prostate biopsy but is self-limiting and rarely (0.6% according to a study [27]) requires surgical intervention or involves an experience of prolonged hematochezia [24]. A large prospective cohort revealed rectal bleeding as one of the common complications, present in 36.8% of patients. Even in this high percentage, only 2.5% reported moderate/major symptomatology [25]. A study reported higher rates of rectal bleeding (more than 10% higher) in men undergoing more biopsy cores (17% for 6 cores vs. 27% for 12 cores) [28]. Massive rectal bleeding is very uncommon, and the management should not be delayed. Rectoscopy is indicated for efficient treatment (vessel clipping, ligation, adrenaline injection, sclerotherapy of rectal balloon) [29].

### 5.4. Erectile Difficulties

One of the most concerning complications for patients is erectile dysfunction which may occur after prostate biopsy. Usually patents completely recover in 1–3 months after biopsy. Because of the heterogenous nature of the data (age, populations, different types of erectile dysfunctions, and significant confounders), it is hard to obtain reliable results [24,30]. A study which used the international index of erectile function (IIEF) score revealed that 34% of patients with no documented erectile dysfunction had a decrease in the IIEF score in 1 week, 20% of them continued to have lower scores at 1 month, and 24% of them even lower scores at 3 months [31]. There was no correlation found in studies between the incidence or duration of the erectile difficulties and the number of biopsied cores [30].

### 5.5. Acute Urinary Retention

Acute urinary retention is a complication with a documented incidence ranging between 0.4% and 6%. Urinary retention has a transient nature, so the placement of a temporary urethral catheter should be enough. Many studies have shown that there is a strong connection between the volume of the prostate, the number of cores taken and the voiding impairment with acute urinary retention [24,32].

### 5.6. Infection 

The infectious complications frequently include urinary tract infections and prostatitis. Although the infection rates vary between low numbers (0.3% and 3.2%), the increases in antimicrobial resistance pose a true concern worldwide [33]. A study conducted between 2017 and 2021 on 784 patents who underwent a TRUS-guided biopsy have shown an overall decrease in the presence of this complication, from 2.7% in 2017 to 0.8% in the first three quarters of 2021 (with a peak of 3.4% in 2018) [34]. Rarer cases might include epididymitis, orchitis, and urinary sepsis. Another study has shown that the risk of infection increases if the patient had undergone a previous biopsy. The highest rate recorded was 15% for patients who had undergone more than 5 biopsies [35].

### 5.7. Sepsis

Sepsis is an important life-threatening reaction triggered by an infection and requires medical assistance immediately. A study conducted on 576 patients who underwent a TRUS-guided biopsy found only 8 cases of sepsis (1% of cases) [36].

## 6. Technical Difficulties

Technical difficulties can be due to the equipment used, the presence of certain pathologies of the patient, and the tumor localization. The following section outlines the most common difficulties that can arise during TRUS-guided biopsy, difficulties that both myself and my colleagues have also encountered in medical practice. These difficulties do not include the absolute contraindications of TRUS (surgical absence of rectum, the presence of a rectal fistula, ilio-anal pouch, inflammatory bowel disease, or severe bleeding diatheses).

### 6.1. Rectal Pathologies

Due to the transrectal approach of the procedure, pathologies such as hemorrhoids (either external or internal) and anal fissures can create difficulties. Both are common anorectal diseases which cause painful defecation (excepting internal hemorrhoids) and anal bleeding [37,38,39]. Rectal bleeding as a complication has been already described, and pain will be detailed in the following subchapter. However, the TRUS-guided biopsy is not an emergency procedure, so patients should first treat the hemorrhoids. Since surgical procedures are painful but quick and effective, some may respond to pharmaceutical treatment. Either way, TRUS should be postponed for a period [38]. Multiparametric-MRI might be a good alternative for immediate results since it has shown promising results, but a biopsy will still be needed for diagnostic confirmation [40].

### 6.2. Prostatic Diameter and Volume

A normal prostate continues to grow from birth until the age of 20. At this age, the size should be 3 cm long, 4 cm wide, and 2 cm in antero-posterior diameter, with a weight of 15–20 g and a volume of 30–40 mL (criteria of a small prostate) [41]. After age 45, the prostate volume becomes unstable and might increase (in some cases even to 100–200 g). A study conducted on 90 patients (age 50–60 years) who underwent TRUS has shown a direct correlation between the volume and the level of total and free PSA in serum [42]. As we here use the 12-core biopsy, a large prostate poses the question of whether 12 cores are enough. Some studies have shown that the risks of an 18-core biopsy might outweigh the benefits, so the 12-core biopsy remains preferred [22]. Another study went further and analyzed the prostate in all three dimensions along with the total volume. All patients had PSA values between 4.0 and 9.9 ng/mL and underwent the 12-core TRUS-guided prostate biopsy. The dimensions were correlated with the anatomopathological diagnosis. The results found that patients with cancer have lower prostate volumes (around 48.85 cc in volume, after which the cancer detection rate began to decrease) [43].

### 6.3. Benign Prostatic Hyperplasia (BPH)

Because tumors are usually multifocal, and their ultrasound appearances have great variability, some of them cannot be differentiated from BPH [44]. Unfortunately, because of these ultrasound similarities, BPH can mask some prostate cancers (especially the lesions located in the central and transitional zone of the gland) [45,46]. In such cases of diagnostic uncertainty, it is best for the patient to undergo additional imaging investigations. Mp-MRI is one solid option in such cases, as transitional zone cancers can be detected with fair accuracy on a T2-weighted signal [45].

### 6.4. Location of the Suspicious Lesion

**Figure 4:**
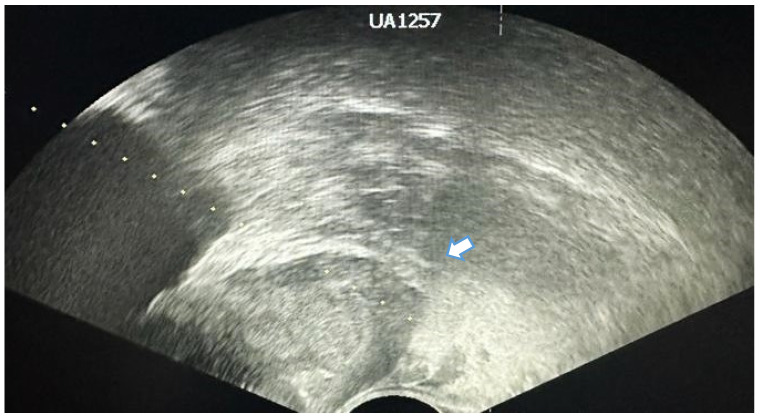
Suspicious hypoechoic areas located at the base of the prostate. White arrow indicating the possible trajectory for biopsy.

**Figure 5:**
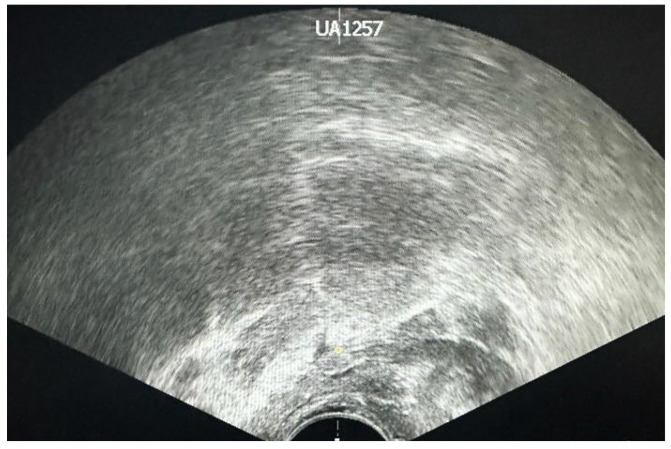
Difficult visualization of anterior-apex lesions because of the depth and the neighboring anatomical structures.

Some lesions are easier to biopsy than others (the base and posterior surface) due to the transrectal approach. The TRUS-guided biopsy gives easy access to the prostate base (Figure 4) but because of the anatomy of the gland, anterior-apex lesions may be difficult to biopsy because of the depth needed to reach them (Figure 5) and because of the urethra which can be punctured in the process [47]. The biopsy of the apical region has great importance since it can increase the overall prostate cancer detection by 7.8% [48]. Some studies recommend sampling the anterior apical region of the prostate using a transperineal approach instead of transrectal, both for easier access and for lower infection risks [49,50].

### 6.5. Prostate Motion

The prostate motion during TRUS-guided biopsy, although small, can be a source of error in puncture alignment. In these cases, the equipment used (2D probe vs 3D probe) is important to reduce the errors and increase the accuracy and efficiency of TRUS-targeted segmentation and biopsy [51]. The use of 3D probes can improve the accuracy of the systematic punctures. Some studies have developed and tested 3D automatic segmentations using AI algorithms, which are clinically feasible and have been validated using manual segmentation (which is the actual gold standard) [51,52].

### 6.6. Pain

The pain is probably the most important obstacle that both the patient and the clinician must overcome, as it can appear in different steps during the whole procedure, from probe insertion and probe manipulation to actual biopsy sampling (multiple punctures with thick needles) and periprostatic possible infiltration. The pain can last several hours after the procedure [24]. A systematic review reported anorectal compliance, lateral decubitus during procedure, the number of cores, prostate volume, and young age to be risk factors predisposing to pain [29].

One study used the visual analog scale (VAS) for post-procedure pain feedback to discover which step patients find most painful. The results showed that most of the patients experienced the greatest pain during the probe insertion in the rectum step [53]. A painful experience can reduce a patient’s compliance and the procedure acceptability. Some studies have shown that the pain during prostatic biopsy is cumulative and directly proportional with the number of cores punctured [54]. More than this, the prostate apex, which we discussed poses some difficulties in reaching, is the most painful site during the biopsy [55].

At first, no pain relief interventions were accepted for TRUS-guided prostate biopsy, but now there are a few modalities available to alleviate and comfort patients’ pain. In literature reviews, there are several papers which have performed trials with different types of anesthesia and placebos [56]. The pain relief methods consist of a periprostatic nerve block (PPNB), local intrarectal anesthetic, and combinations of the two [54].

The PPNB has shown great results, greatly reducing the pain during the procedure, compared to patients who did not have anesthesia at all [57]. There are several sites available for prostatic nerve block from which the binasal injections (between the prostate base and each seminal gland) are the most common [58]. In addition to patients becoming more cooperative and accepting another biopsy (if necessary), PPNB also reduces the post-procedure need of analgesics [54]. A study conducted on overall morbidity after TRUS-guided prostate biopsy showed that from 405 patients (which underwent the standard 12-core procedure under PPNB with 10 mL of 1% lidocaine and gave responses to questionnaires), 63% fully recovered from the pain, 35% declared minimal discomfort, and only 2% requested assistance for daily needs [59].

The intrarectal local anesthetic is easily absorbed in the rectal wall and can reduce the biopsy pain, but is not as efficient as PPNB. The probe insertion pain does vary, as more factors can contribute to the efficiency of the local anesthetic (the type of agent used and thickness of the probe). The intrarectal local anesthetic, however, has not shown any statistically significant results in pain reduction when compared with patients with no anesthesia [56,60].

Combined anesthesia (PPNB with intrarectal local anesthetic) has become more and more popular recently. It aims to block all sensory nerves from the region, as prostatic nerve does not control the rectum pain. The combination of the two proves to be superior to any of the techniques used alone. Proof of an increase in the overall complication rate was not found [54,61,62].

The complications of the PPNB are unusual and do not increase or contribute to the earlier mentioned complications of prostate biopsy. The pain due to needle puncture for PPNB anesthesia is the most frequent side effect, but it is a good tradeoff since the actual biopsy will be painless. In rare cases, PPNB might cause vasovagal syncope [63,64].

## 7. Discussion

TRUS-guided prostate biopsy has been for decades the main way to diagnose prostate cancer [65]. As the transperineal prostate biopsy has become more widespread, lists of pros and cons have been made regarding both transrectal and transperineal procedures. The advantages of transrectal biopsy are mostly related to the cost-effectiveness (in terms of time invested, supplies used, and being widely available) [19]. One of the major disadvantages is the high false-negative rate which can occur due to the operator’s experience and the lack of constant visual contact, which leads to an unequal distribution of biopsy cores [66]. With experience and practice, this limitation decreases. A simulator study has proposed a real-time 3D simulator to prepare and train young urologists to keep the probe pitch neutral during a side-fire prostate biopsy. The results have shown that the technique can be learned by the participants (the program detected the urologists’ improvements when compared to template deviation), suggesting that it may also reduce the number of false-negative prostate biopsy results [67]. The second one refers to the need for antibiotic prophylaxis and the risk of infection and sepsis. The colon is rich in microbiota and puncturing the colon wall can inseminate bacteria in the prostate [68]. As described earlier, the infectious complications rates vary. A study has compared more articles and found the infections rates to be between 1% and 17.5% [24]. Because of the recent fluoroquinolone-resistances of bacteria, more serious infections complications have arisen, which require hospitalization [69]. A recent study compared the efficiency of ciprofloxacin versus ciprofloxacin in combination with Fosfomycin, in cases of urosepsis post-TRUS-guided prostate biopsy. The conclusion of the study was that the prophylaxis combination of ciprofloxacin with Fosfomycin significantly lowered the urosepsis rates [70].

Another important aspect of the TRUS is the social impact of the procedure. The role of prostate biopsy has changed from pure cancer detection to overall management of the patient [71]. The pain, the possible complications, and the result definitely have an impact on patients undergoing this procedure. The most frequent psychological symptoms found in studies are anxiety and depression [72]. In a study, 114 patients were given reports to answer some questions on the perceived stress, the generalized self-efficacy, and sense of coherence after puncture. The results have shown that perceived stress was the strongest predictor of emotional adjustment and mood disturbance [73]. Also, anxiety (especially in younger patients) is an important factor in pain, since pain is subjective, and anxiety can lower a patient’s threshold [24]. Another study investigated the link between the pre-procedural waiting period and the level of anxiety and pain perception. The conclusion was that in patients with high levels of anxiety, it is best to perform the biopsy as soon as possible to obtain a positive impact on patient tolerance [74]. By communicating and improving criteria for patients in need of prostate biopsy, morbidity and psychological impact can also be improved [71,72].

Although many clinicians prefer the combined anesthesia (PPNB with intrarectal local anesthetic), new studies have searched for alternative anesthetics. A methoxyflurane inhaler appears to be a safe and effective method of analgesia [75,76]. Although the methoxyflurane did not improve the immediate pain scores, it did improve the overall experience and the willingness of patients to repeat the TRUS prostate biopsy if necessary [77]. Another study found tramadol to be an excellent and safe analgesic adjunct to PPNB. Tramadol’s effectiveness can be increased even further by the simultaneous administration of parecoxib [78].

The dynamic contrast enhanced power Doppler has been explored as an imaging technique for prostate cancer detection. Unfortunately, only the lesions localized in the right or the left side of the gland could be localized (with an accuracy of 78%), while the malignancies in the ventral and dorsal sides of the prostate could not be assessed [79]. Thus, the dynamic contrast enhanced power Doppler is unlikely to become a future non-invasive prostate cancer detection tool, but it could improve the detection of zones which should be punctured during biopsy according to a study [80].

Multiparametric ultrasound (MpUS)-MRI-TRUS fusion biopsy is receiving increasing attention because of the valuable information of the prostate cancer foci that it generates. The combination of MpUS with the MpMRI-TRUS fusion can overcome the clinical detection limitations of each imaging modality through the real-time lending of the images [81]. There are several software available that perform the real-time fusion, although in certain centers the images are mentally superimposed by the operator that is performing the biopsy [82]. This fusion procedure can detect prostate cancer at higher rates than normal ultrasound prostate biopsy, easily identifying even the index lesions with extra-prostatic extension [83]. The MpMRI in prebiopsy lesions has a sensitivity of 93% but a specificity of only 41% to detect cancerous lesions. Compared with the TRUS-guided biopsy’s low sensitivity (of 48%) and high specificity (of 96%), the (MpUS)-MRI-TRUS fusion biopsy should cover both the sensitivity and the specificity [9].

In terms of pain, a study which compared the pain experienced by patients who underwent the standard TRUS 12-core prostate biopsy versus patients who underwent MpMRI-guided fusion prostate biopsy showed no significant difference between the two groups, although the fusion biopsy is usually performed through a perineal approach [53]. Another advantage that MRI-TRUS fusion poses is that it can be used for brachytherapy (which consists of implanting radioactive seeds in the prostate gland), thus transforming a diagnosis procedure into a safe, accurate form of treatment for localized prostate cancers [84]. Recent studies have shown that multiparametric ultrasound-targeted biopsy has obtained favorable results compared to (MpUS)-MRI-TRUS fusion targeted biopsy in patients with a risk of prostate cancer [85].

With so many possibilities for diagnostic approaches, the question of cost-effectiveness has been raised. A study compared the standard TRUS-guided biopsy (which often occurs in the physician’s office under local anesthesia) with the transperineal template biopsy (under general anesthesia), MRI-TRUS fusion biopsy (under sedation), and in-bore MRI biopsy (under sedation). The conclusion of the study has revealed that the additional imaging and sedation/general anesthesia, along with the transperineal template, increase the costs significantly compared with the standard TRUS-guided biopsy, but sometimes these costs are worth the effort to increase the diagnostic accuracy [86].

A recent study has compared the outcomes (in terms of prostate cancer detection) of the TRUS-guided standard 12-core biopsy and MpMRI-targeted 12-core biopsies, with an artificial intelligence program (AIUSP) made to assist a 6-core targeted biopsy. The data set consisted of 400 patients, distributed equally between procedure types (133 patients for each of TRUS and AIUSP groups and 134 for the MpMRI group). The results showed that the highest detection rate of prostate cancer was achieved by AIUSP (with an accuracy of 49.6%) [87]. In future, artificial intelligence assistance might be the key to increasing the diagnostic accuracy even for the TRUS which is considered a low-performance modality [88].

Over the last decade, a great number of articles have been published regarding the importance of PSMA PET-CT in both the primary diagnosis of prostate cancer as well as in recurrences that might occur. For the moment, PSMA PET/CT has a higher importance in recurrences, when the diagnosis is known, because of the consistent number of pathologies that can express PSMA receptors. In addition to the great cost of this diagnostic method, there must be a strong collaboration between the urologist and the nuclear medicine doctors, and comprehensive knowledge of the various influencing factors must be studied to obtain the best results [89].

## 8. Strengths and Limitations

The strengths of the study include the review of both technical difficulties and post-procedural complications in the same article, also providing information on how to overcome these events. 

For the limitations, the study is not a meta-analysis, so the selecting criteria were not too strict. Because of limited access, some interesting and relevant articles had to be discarded, although they probably would have not changed this paper’s conclusions. Finding articles on technical difficulties during TRUS-guided prostate biopsy has proven to be a difficult task, since direct keywords did not return adequate results.

## 9. Conclusions

TRUS-guided prostate biopsy remains a solid, cost-efficient, and safe procedure to diagnose prostate cancer. The complications are usually self-limiting and do not require additional medical assistance. The difficulties posed by the procedure can be safely overcome if there are not any other available alternatives. Open communication with the patients improves both pre- and post-procedure compliance.

## Data Availability

All data are available from the corresponding authors on request.
